# Coping with DNA Double-Strand Breaks via ATM Signaling Pathway in Bovine Oocytes

**DOI:** 10.3390/ijms21238892

**Published:** 2020-11-24

**Authors:** Lili Wang, Xiaolei Xu, Mingming Teng, Guimin Zhao, Anmin Lei

**Affiliations:** 1 Shaanxi Stem Cell Engineering and Technology Research Center, College of Veterinary Medicine, Northwest A&F University, Yangling 712100, China; iamwanglili@nwafu.edu.cn (L.W.); xuxiaolei@nwafu.edu.cn (X.X.); tengmingming@nwafu.edu.cn (M.T.); 2 Key Laboratory of Infection and Immunity of Shandong Province, Department of Immunology, School of Biomedical Sciences, Shandong University, Jinan 250012, China; zhaoguimin@sdu.edu.cn

**Keywords:** DNA double-strand breaks, *ATM*, *p21*, bovine oocyte, in vitro maturation

## Abstract

As a common injury almost all cells face, DNA damage in oocytes—especially double-strand breaks (DSBs), which occur naturally during the first meiosis phase (meiosis I) due to synaptic complex separation—affects the fertilization ability of oocytes, instead of causing cancer (as in somatic cells). The mechanism of oocytes to effectively repair DSB damage has not yet been clearly studied, especially considering medically induced DSBs superimposed on naturally occurring DSBs in meiosis I. It was found that maturation rates decreased or increased, respectively corresponding with overexpression or interference of *p21* in bovine oocytes. At the same time, the maturation rate of bovine oocytes decreased with a gradual increase in Zeocin dose, and the *p21* expression in those immature oocytes changed significantly with the gradual increase in Zeocin dose (same as increased DSB intensity). Same as *p21*, the variation trend of *ATM* expression was consistent with the gradual increase in Zeocin dose. Furthermore, the oocytes demonstrated tolerance to DSBs during meiosis I, while the maturation rates decreased when the damage exceeded a certain threshold; according to which, it may be that *ATM* regulates the p53–p21 pathway to affect the completion of meiosis. In addition, nonhomologous recombination and cumulus cells are potentially involved in the process by which oocytes respond to DSB damage.

## 1. Introduction

The maturation and quality of oocytes form the basis of fertility in all mammal species, which can be easily disturbed by many factors. The evaluation of oocyte morphological characteristics is very important for accurately predicting oocytes and the subsequent embryonic developmental potential. Besides oocyte shape, the cytoplasm and its contents, perivitelline space, zona pellucida, the morphology of the PBI (the first polar body), spindle morphology, follicular fluid, and cumulus cells are the main terms considered [1,2,3,4,5,6,7,8]. In addition, some molecular-level detection indicators may also reflect the quality of oocytes, such as *GDF9* (growth and differentiation factor 9), *BMP15* (bone morphogenetic protein 15) [9,10,11,12,13], *FSHR* (follicle-stimulating hormone receptor) [14,15], *H1foo* (oocyte-specific histone 1) [16,17], *cyclin B* [18,19], and so on.

As a common injury, almost all cells face DNA damage. In oocytes, DNA damage does not cause cancer but, instead, affects their fertilization ability. Mammalian oocytes with proliferating capacity transform into primary oocytes before they are released, being arrested at the double-line phase in meiosis I, which is maintained for many years (even ≥40 years in humans) until ovulation after stimulation by hormones. Therefore, the oocytes of some species experience a certain timeframe, perhaps even comprising decades, of damage accumulation. In this case, some effective DNA damage recognition and repair mechanisms are required by the body in order to ensure that healthy oocytes are bred in the ovaries. If DNA damage in oocytes cannot be repaired in a timely and effective manner, the failure of normal maturation and fertilization of oocytes may occur, resulting in offspring with diseases or mutations [20,21,22]. However, the molecular mechanisms by which oocytes control and effectively repair these injuries have not yet been clearly elucidated.

It has recently been discovered that DSBs at the G2 phase of meiosis in mouse oocytes did not prevent them from entering MI (the metaphase of meiosis I) unless the damage was particularly severe. The possible reason for this is a lack of effective DNA damage checkpoints (DDCs), leading to the ataxic telangiectasia mutation (ATM) kinase—the main regulatory factor in the DNA damage response pathway—not being effectively activated in oocytes [22]. Once activated in G1/S, S or G2/M transition periods, ATM phosphorylates a series of substrates to regulate the cell cycle. The adjustments occur at multiple levels in order to maintain the stability of checkpoints. In the absence of ATM, the stability and efficiency of p53 (tumor protein 53) decrease. ATM directly phosphorylates Ser-15 and many other sites of p53, which are only related to its efficiency and has no effects on its stability. ATM also phosphorylates three other proteins, Chk2 (checkpoint kinase 2), Mdm2 and Mdmx, which may have impacts on the stability of p53 at the G1/S transition checkpoint [23]. When activated, p53 triggers the p21/WAF1 pathway, which affects the activity of cyclin kinases and blocks cell progression from the G1 phase to the S phase [24].

Activity changes of MPF (maturation-promoting factor, mitosis-promoting factor, M phase-promoting factor or CDK1-cyclin B1) play a key role in the regulation of meiosis in mammalian oocytes. Cyclin-dependent kinase inhibitor 1A (*p21*, *Cip1*, *CDKN1A*) was the first Cip/Kip family member discovered, which plays the role of a CKI (cyclin-dependent kinase inhibitor). It has been confirmed that p21 can directly inhibit the phosphorylation of CDK1 (cyclin-dependent kinase 1) at Thr-161, thereby inhibiting MPF activity in lower animal eggs [25]. However, to the best of our knowledge, the role of *p21* in the regulation of meiosis in bovine oocytes has not yet been reported.

In this study, we determine the important role of *p21* in bovine oocyte maturation by overexpression or interference of *p21* and, accordingly, zeocin is used to manufacture DNA DSBs in bovine oocytes. KU-55933, a specific inhibitor of ATM, is also used. In the case of DNA DSBs, the expression of *p21* was assumed to be increased with the damage intensity in immature oocytes, which was in correspondence with the maturation rates, while the relative expression trend of *ATM* was similar to that of *p21*. When ATM was inhibited, the maturation rates were decreased. Furthermore, oocytes responding to damage may be involved in nonhomologous recombination and cumulus cells. The expression of *p21* and oocyte quality assessment genes were further detected in order to explore their correlation and whether *p21* can be used as a reference for oocyte quality assessment.

## 2. Results

### 2.1. Location of p21-Venus in HeLa Cells

**Figure 1:**
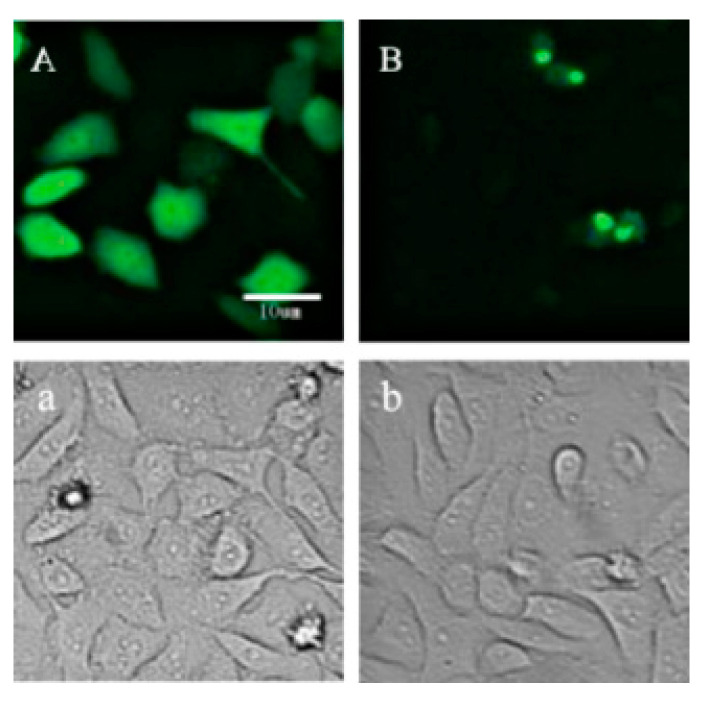
Expression of fluorescence proteins of Venus and *p21*-Venus after transfection in HeLa cells. (**A**,**a**): HeLa cells transfected with Venus; (**B**,**b**): HeLa cells transfected with *p21*-Venus. Scale bar = 10 μm.

The amplification and validation information of *p21*-Venus fusion plasmid is shown in Figure S1. To further validate the expression of *p21*-Venus, we transfected the plasmid into HeLa cells before microinjection. As observed by fluorescence microscopy, the Venus vector and *p21*-Venus fusion plasmid could both be effectively expressed in HeLa cells. The results showed that the green fluorescence of Venus was distributed throughout the cells, not showing any clear localization (Figure 1A,a). The green fluorescence of *p21*-Venus in HeLa was mainly concentrated in the nucleus (Figure 1B,b). The above results indicate that *p21* was expressed normally in Hela cells. Therefore, in subsequent experiments, we used *p21*-Venus to overexpress p21 and to determine its biological effects on the IVM of bovine oocytes.

### 2.2. Effects of p21 on Oocyte IVM

**Figure 2:**
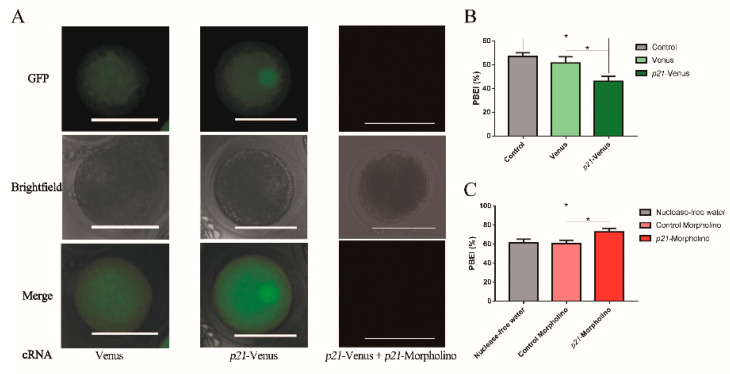
Effects of *p21* on oocytes: (**A**) cRNA microinjection of Venus or *p21*-Venus and co-injection with *p21*-Venus cRNA and *p21*-Morpholino in oocytes under Fluorescence microscope; scale bar = 100 μM; (**B**) the effects of *p21* overexpression on the PBEI(%); and (**C**) the effects of *p21* interference on the PBEI(%). * indicates *p* < 0.05.

To observe the location of *p21* in oocytes, the cRNAs of Venus and *p21*-Venus were microinjected into GV-stage oocytes. The cRNA concentrations and ODs of *p21*-Venus and Venus are shown in Table S1. Following 3 h of culture, the oocytes were imaged by fluorescence microscopy. After injection of Venus cRNA, green fluorescence was seen to be distributed throughout the whole of oocytes. Green fluorescence of p21-Venus was also distributed throughout the oocytes after microinjection with *p21*-Venus cRNA, where the fluorescence intensity in and near the nucleus was obviously stronger than other cytoplasm sections (Figure 2A), similar to the localization results in HeLa cells (Figure 1). These results indicate that *p21*-Venus cRNA can be highly expressed in oocytes with specific localization and, so, *p21* may play an important role in bovine oocytes.

To further detect the effects of *p21* on oocyte maturation, *p21*-Venus cRNA or *p21*-Morpholino were microinjected into oocytes for overexpression or interference of *p21*. The PBEI(%) was calculated at 22 h. First, the efficiency of *p21*-Morpholino was verified. By co-injection with *p21*-Venus cRNA and *p21*-Morpholino, we found that the fluorescence intensity in the *p21*-Venus cRNA injection group was obviously stronger than that in the *p21*-Venus cRNA and *p21*-Morpholino co-injection group after 3 h of culture (Figure 2A). At 22 h, the PBEI% of the *p21*-Venus group decreased by about 10%, compared with the Venus group and the control group (Figure 2B). On the contrary, the PBEI% of *p21* interference (the *p21*-Morpholino group) was improved by about 12%, compared with the control group of meaningless interference segments and nuclease-free H_2_O (Figure 2C).

### 2.3. Effects of DNA DSBs Induced by Zeocin on Bovine Oocyte IVM

Oocyte meiosis is often accompanied by DNA DSBs and reconstruction, which occur naturally during meiosis I due to the synaptic complex separation. In view of the above research results regarding *p21* in oocyte maturation and its correlation with DNA damage, we wanted to explore the potentially related mechanisms during oocyte IVM after DNA DSBs. First, HeLa cells were used to delineate suitable concentrations of Zeocin inducing DNA DSBs. We found that the cell proliferation was significantly reduced at the maximum dose of 2.0 μg/μL of Zeocin, according to the dosage instructions from Invitrogen (Beijing, China) (Figure S2A) and, so, we used 2.0 μg/μL (≈1758.38 μM) of Zeocin as the maximum dose to construct the DNA DSBs model of bovine oocytes.

**Figure 3:**
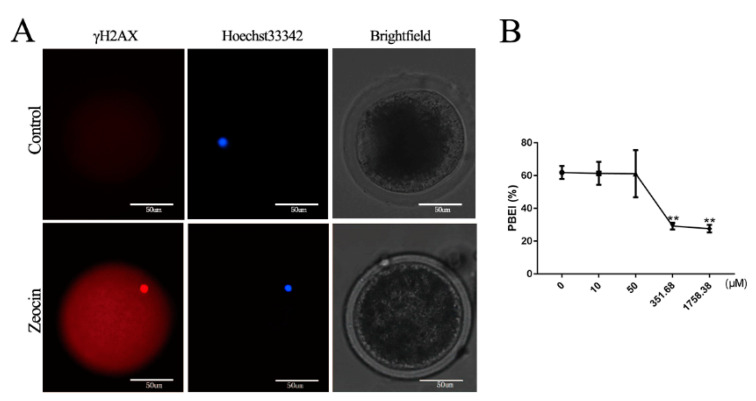
Effects of DNA double-strand breaks (DSBs) induced by Zeocin on bovine oocyte *in vitro* maturation (IVM): (**A**) immunofluorescence of γH2AX in the oocytes after bovine cumulus–oocyte complexes (COCs) treated with 10 μM Zeocin for 2 h, scale bar = 50 μM; (**B**) polar body excretion (PBEI) (%) of oocytes after bovine COCs were treated with different concentrations of Zeocin (10, 50, 351.68 or 1758.38 μM) for 22 h. ** indicates *p* < 0.01.

Based on the above test results, we set up several concentration gradients (0, 10, 50, 351.68 or 1758.38 μM Zeocin; Table S1B) to investigate the effects of DSBs induced by Zeocin on oocytes. At 2 h, the fluorescence of γH2AX was observed in the group with a minimum concentration of Zeosin (10 μM), indicating that DSBs had formed successfully (Figure 3A). In parallel, the Dos and COCs were placed in maturation medium to continue culturing for another 20 h after treatment with Zeocin for 2 h. At 22 h, there was no significant change in the maturation rates (PBEI%) in low concentration Zeosin (i.e., 10 μM and 50 μM) groups, compared with the control, while the PBEI% decreased significantly (*p* < 0.01) after Zeocin reached a certain concentration (i.e., 351.68 μM and 1758.38 μM groups; Figure 3B).

### 2.4. Expression of DSB Repair Genes in Oocytes Was Altered under Different Zeocin Concentrations

**Figure 4:**
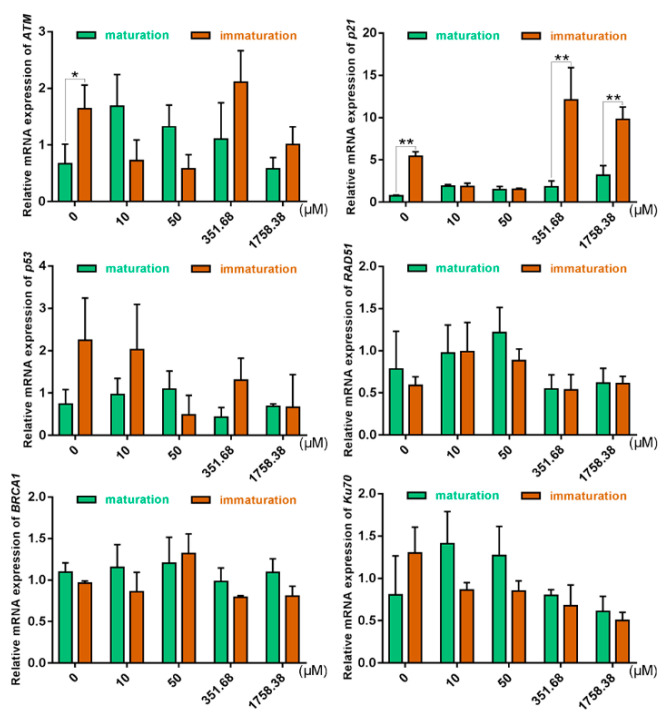
Relative mRNA expression levels of DNA DSBs repair genes in oocytes by qRT-PCR. After bovine COCs were treated with different concentrations of Zeocin (0 μM, 10 μM, 50 μM, 351.68 μM or 1758.38 μM) for 22 h, the oocytes at PBEI (“maturation”, green charts) and oocytes at GV (“immature”, orange charts) were collected to detect DNA DSBs repair genes. * indicates *p* < 0.05, ** indicates *p* < 0.01.

Our results (Figure 4) show that the relative mRNA expression level of *p21* in immature oocytes was insensitive under low concentrations of Zeocin (10 μM and 50 μM); however, as the concentration of Zeocin increased, the expression level of *p21* in immature oocytes was raised. In the control group and high-concentration Zeocin (351.68 μM and 1758.38 μM) groups, higher expression levels of *p21* in immature oocytes than that in mature oocytes were shown, where the trend of change was consistent with the maturation rates of oocytes (i.e., when *p21* was overexpressed, oocyte maturation was inhibited; Figure 2B). In the low-concentration Zeocin (10 μM and 50 μM) groups, *p21* expression levels were similar between immature and mature oocytes, where the expression level of *ATM* was the same as that of *p21* in immature oocytes. In this situation, it was likely that the function of *ATM* was not to repair DSBs, but, instead, to play a cell cycle arrest function in the p53–p21 pathway.

### 2.5. The Effects of Cumulus Cells on the Response of COCs to DNA DSBs in Prophase of Meiosis I

Cumulus cells are very important for oocytes during the prophase of meiosis I. According to the effects of different concentrations of Zeocin on the maturation rates (PBEI%), we found that there was no significant difference under the concentrations of 10 and 50 μM, compared with the control (Figure 3B). They may have been closer to physiological DSB conditions than with 351.68 or 1758.38 μM Zeocin. We attempted to explore the molecular mechanism of cumulus cells on the response of COCs to DNA DSBs in prophase of meiosis I, where the conditions are closer to physiological conditions and have more severe damage.

**Figure 5:**
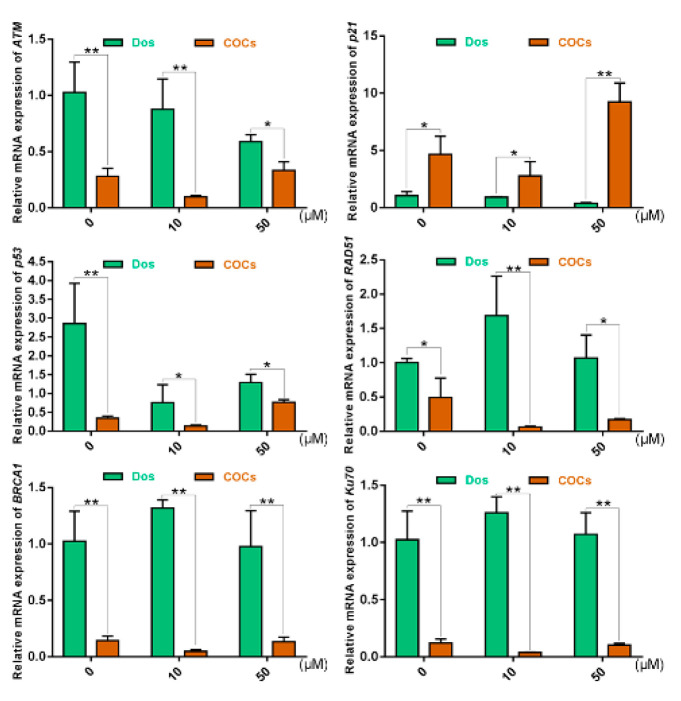
Relative mRNA expression levels of genes in oocytes by qRT-PCR after bovine denuded oocytes (Dos) and COCs treated with different concentrations of Zeocin (10 μM and 50 μM) for 2 h. * indicates *p* < 0.05, ** indicates *p* < 0.01.

Our results showed that, in each group, DNA repair-related genes (*ATM*, *p53*, *RAD51*, *BRCA1* and *Ku70*) in Dos presented higher expression levels compared to those in COCs (Figure 5). This demonstrated the lower sensitivity to DNA DSBs induced by Zeocin in COCs, from which it can be inferred that COCs have a higher resistance to DSB damage and that cumulus cells may buffer the damage of Zeocin on oocytes. We believe that the cumulus cells protected oocytes from the damage to some degree; seriously, the fate of cumulus cells would come to apoptosis. *p21* in Dos showed lower expression than in COCs. In addition, the change trends of *p21* and *p53* in bovine oocytes treated with Zeocin were basically consistent.

### 2.6. Oocyte Maturation and Gene Expression Altered with ATM-Specific Inhibitor

The above results indicated that *ATM* and cumulus cells might play key roles during the maturation of oocytes. We decided to explore the roles and mechanisms of *ATM* in oocyte quality and cell cycle control. Therefore, the ATM-specific inhibitor KU-55933 was used during IVM of bovine oocytes, which effectively acted on ATM-dependent phosphorylation [26,27].

**Figure 6:**
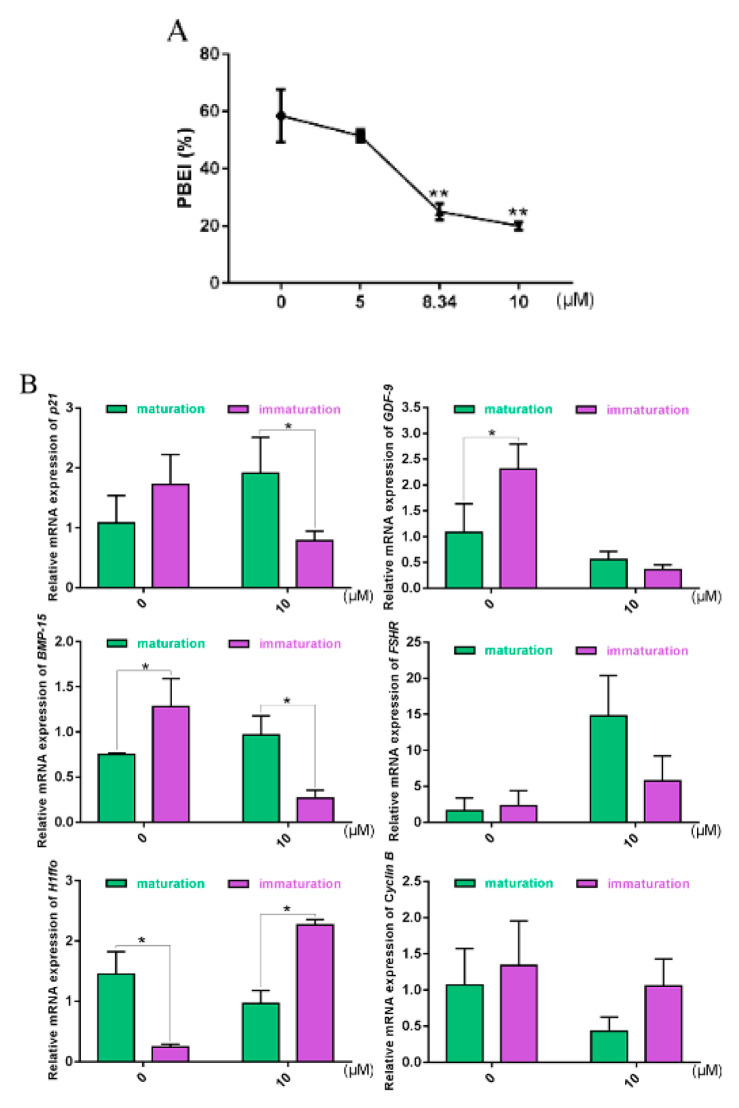
Effects of the ataxic telangiectasia mutation (ATM)-specific inhibitor KU-55933 on bovine COCs: (**A**) PBEI% of bovine COCs with different concentrations of KU-55933 treatment for 22 h; (**B**) after bovine COCs were treated with 10 μM KU-55933 for 22 h, the oocytes at PBEI (“maturation”, green charts) and oocytes at GV (“immature”, purple charts) were collected, in order to detect the genes for oocyte quality evaluation and cell cycle. * indicates *p* < 0.05, ** indicates *p* < 0.01.

Our results showed that the maturation rates of bovine oocytes decreased significantly with increased concentrations of KU-55933 (Figure 6A), indicating that *ATM* likely plays an important role during PBEI of bovine oocytes.

According to previous studies, *p21* shows higher expression in immature oocytes than mature oocytes under normal culture conditions. Under the action of KU-55933 (Figure 6B), *p21* in immature oocytes tended to be downregulated. As oocyte-secreted factors, *GDF9* and *BMP15* have been shown to play key roles in modulating both the cell fate of the somatic granulosa cells and the quality and developmental competence of oocytes [9,10,11,12,13,28]. *FSHR*, the receptor of FSH (follicle-stimulating hormone), is typically involved in mammalian reproduction. The function of FSH is performed upon it binding to its cognate receptor, FSHR, expressed on the surface of target cells (e.g., granulosa and Sertoli cells) [14,15]. *H1foo*, a unique sub-type, can only be found in oocytes of different organisms [16,17]. *Cyclin B*, a maternally derived gene, is involved in cell cycle regulation and plays an important role during oocyte maturation [18,19]. Our results showed that *GDF-9* and *BMP-15* were downregulated in immature oocytes under the action of KU-55933, compared with the control, while their trend was contrary to that of *H1foo*.

## 3. Discussion

It is well-known that the quality of embryos and the origins of oocytes are strongly related, while the cleavage rates are determined by both the oocytes and sperm [29]. Even so, oocytes are still the main determinant of embryonic development potential, which provide half of the embryo’s chromosomes and almost all cytoplasmic components. Therefore, in terms of impact on the embryo, oocytes bear much more responsibility than sperm. The identification and screening of oocyte quality have become the key to affecting human-assisted reproduction and animal cloning [30]. DNA damage in mammalian oocytes was first discovered during meiotic recombination. Meiotic recombination occurs from the leptotene stage to the pachytene stage of meiosis prophase before birth, where the natural formation of DSBs is included [31,32,33,34]. In clinical medicine, some cancer patients undergoing chemotherapy still hope to have a normal pregnancy; however, chemotherapeutic drugs often trigger DSBs. In female reproduction, DNA damage may lead to infertility and even genetic abnormalities, which may be transferred to the embryos [35,36]. Through DNA damage response (DDR), cells can ensure their normal survival and the stability of the genetic genomes, simultaneously preventing cancer. Cell cycle arrest is one mechanism by which cells respond to two different types of DNA damage: single-strand breaks (SSBs) and DSBs. The purpose of the mechanism is to set aside time for cells to repair the damage. Therefore, DDR is involved in two processes—one of which is the activation of DDCs, the other of which is the repair mechanism for damage. As the repair mechanism is closely related to the cell cycle, the cell cycle should immediately return to normal once the DNA damage has been repaired. In turn, if damage-causing events prevent the complete repair of DNA damage, apoptosis-related pathways should be activated to clean up the damaged cells and prevent the potential canceration, which may be subsequently triggered [37,38].

A previous study has shown that DNA damage, specifically DSBs, can lead to *p21* overexpression and triggering of the p53–p21 response, while *p21* inhibition exacerbates the frequency of apoptosis in beta-cells [39]. As an inhibitor of cyclin-dependent kinases (CDKs), *p21* has been shown to impair cell cycle progression in the G1-to-S phase by regulating cell cycle checkpoints [40]. Therefore, we first used the *p21*-Venus eukaryotic expression vector in our laboratory [41,42] and *p21*-Morpholino to explore the possible mechanisms of bovine oocytes responding to DSBs, in order to provide a reference for the nondestructive testing of oocyte quality assessment. After the interference of p21, the maturation rate of oocytes increased, which was consistent with our previous studies [41,42]. Some oocytes with *p21* interference could not successfully extrude the PBI. Previous studies on *p21* involving the regulation of meiosis in mammals have mainly focused on the direct inhibition of *p21* on CDK1 kinase phosphorylation [43], thereby inhibiting MPF activity [44]. Here, we speculate that *p21* may carry out the function of screening oocytes: In the presence of *p21*, parts of oocytes with “defects” were arrested, becoming unable to normally extrude the PBI and, thus, were eliminated. Specifically, it is worth thinking about which part of the oocytes with “defects” passed through the screening and extruded the PBI after *p21* was disturbed?

We speculated that there also exists a certain relationship between *p21* and DNA DSBs in the mechanisms that affect oocyte maturation. It has recently been discovered that DSBs at the G2 phase of meiosis in competent mouse oocytes cannot prevent them from entering the MI phase unless the damage was particularly severe. The possible reason for this was the lack of effective DDCs in oocytes, thereby leading to ATM kinase not being effectively activated [22]. In our study, *p21* and genes of the ATM pathway related to DSB repair were detected and, so, a specific ATM inhibitor (KU-55933) was used in combination [45]. Our results showed that the maturation rates did not change significantly at low concentrations of Zeocin (10 μM and 50 μM), which may be due to a lack of effective DDCs. As further evidence, the insensitivity of DDCs in oocytes was found to be due to the decrease of ATM activity in the study of Marangos [22]. Similarly, immature oocytes were also insensitive to changes in *p21* expression under low concentrations of Zeocin (10 μM and 50 μM; Figure 4), while the *ATM* expression at the corresponding concentration of Zeocin was relatively low, consistent with the results of Marangos [22]. However, there was a decrease in the maturation rate of oocytes under high-concentration Zeocin (351.68 μM and 1758.38 μM) treatment (Figure 3B). Similarly, it has been shown that DSBs can inhibit the process of meiosis directly and that the meiotic maturation of oocytes is sensitive to DSBs [46].

Due to the prolonged stasis of the meiosis prophase, oocytes are extremely vulnerable to the accumulation of environmental damage, including DNA damage [22]. In addition, cumulus cells affect the maturation, ovulation and even fertilization of oocytes [47]. In turn, oocytes also regulate the extracellular matrix, steroid hormone synthesis, active metabolism and cell differentiation of cumulus cells [48]. ATM is one of the major regulators of DNA damage, which can phosphorylate the histone H2AX, generating the epitope recognized by anti-γH2AX antibodies [46,49,50,51,52]. In view of its critical role played after DNA damage, *ATM* interacts with a wide range of protein networks, including p53–p21, resulting in G1 arrest and *RAD51* and *BRCA1* associated with DNA repair [53]. Ku70 is another protein involved in DNA repair. Ku70 and Ku80 make up the Ku heterodimer, which binds to DNA DSBs ends and is required for the nonhomologous end-joining (NHEJ) pathway of DNA repair [54,55]. Compared with COCs, DNA repair-related genes (*ATM*, *p53*, *RAD51*, *BRCA1* and *Ku70*) in the Dos of all groups showed higher expression levels. This indicated stronger resistance to Zeocin-induced DNA damage in COCs, thus demonstrating the important role of cumulus cells in buffering damage.

Oocyte quality has always been an important evaluation criterion in various fields. Through autocrine and paracrine mechanisms, the GDF-9 and BMP-15 system has been shown to play vital roles in oocyte development, ovulation, fertilization and embryonic competence by regulating the growth, differentiation and function of granulosa and thecal cells during follicular development [9,10,11,12,13]. *H1foo* has previously been reported to be restricted to existing in growing/mature oocytes and zygotes [16]. *H1foo* is involved in various mechanisms, such as chromatin organization and gene transcription. With its location on chromatin, *H1foo* is not only involved in chromatin conformation but also participates in the activation or repression of genes during oogenesis and embryo development, before embryonic genome activation [17]. FSHR is a receptor for FSH, which plays a key role in reproduction, including male sperm production and female estrogen production [14,15]. In the meiosis regulation of mammalian oocytes, MPF activity changes play a key role. The *p21* protein was the first member of the Cip/Kip family to be discovered and confirmed to have CKIs. In lower animal eggs, *p21* has been shown to directly inhibit the phosphorylation of Thr-161 of CDK1 kinase, thereby inhibiting MPF activity [56], while the role of *p21* in the meiosis regulation in mammalian oocytes has not yet been reported. Our results showed that the maturation rates of bovine oocytes decreased significantly with an increase in the concentration of ATM inhibitor KU-55933. This indicates that *ATM* plays an important role in the PBEI of bovine oocytes. Consistent with previous research results, *p21* showed higher expressions in immature oocytes than mature oocytes under normal culture conditions. Under the action of KU-55933, *p21*, *GDF-9*, and *BMP-15* in immature oocytes had downward trends (Figure 6B). Studies have shown that the rates of GVBD (germinal vesicle breakdown) decreased after *ATM* inhibition, while the mRNAs of *BMP-15* and *GDF-9* were also downregulated [49,50]. Studies regarding *p21* have previously focused on MPF inhibitors. It has also been recently reported that *p21* promotes DNA DSBs repair [57]. It is not simply through the upregulation of *p21* when somatic cells encounter DNA DSBs: *p21* was also shown to be upregulated after mild DNA damage, while there was brief downregulation of *p21* after violent DNA damage. The reason for this brief downregulation was the presence of Chk1 (checkpoint kinase 1), which phosphorylated the residues of Thr-145 and Ser-146 of *p21*, triggering protease degradation. Later compensation was due to the accumulation of p53-dependent *p21*, activated by the ATM-Chk2 pathway. The degradation of *p21* activated the apoptosis program, thus clearing irrecoverable cells [58].

In conclusion, we make a bold conjecture: The tolerance of oocytes to DNA DSBs during meiosis I was not unfettered, but, by homologous recombination, to withstand low-intensity damage; while, when reaching the threshold of damage to disable the repair function, the cell cycle process is arrested by ATM acting on p53–p21, thereby preventing oocytes from completing meiosis I, such that the maturation rates declined at this time. Simultaneously, there was also a change in oocyte quality. In addition, COCs were more resistant to DNA DSBs agents than Dos, thus indicating that cumulus cells may play an important role during the prophase of meiosis I. It may be that the maturation of oocytes is common, while the immaturation of oocytes is a comprehensive result due to multiple reasons.

### Preconception

(1) During meiosis I, *Ku70*, a key gene of nonhomologous recombination, was involved in coping with DNA damage when it did not reach the threshold of damage to arrest the cell cycle; after the damage reached the threshold and could not be dealt with completely by the repair function, the cycle process was arrested and the completion of meiosis I was prevented.
(2) When GVBD, physiological DNA SSBs, and DSBs form during the displacement and separation of chromosomes, the fractures of damaged DNA need to be repaired quickly in order to ensure the successful completion of the subsequent maturation process in oocytes. Carroll et al. also confirmed that the expression of the DNA damage marker protein γH2AX increased 6-fold after GVBD, compared with the GV phase [22]. When abnormal conditions lead to repair obstacles by physiological DNA SSBs or DSBs damage, is there a DNA damage check mechanism in oocytes at this time?

By studying the DNA damage response of mouse oocytes, Carroll et al. believed that the fully grown mouse oocytes at the G2/M conversion stage lacked an effective DNA damage response mechanism [22]. Sun et al. found, in the meiotic study of mouse oocytes, that the overexpression of Chk1 before GVBD could block the division process by Cdh1 and cyclin B pathways, as well as activating the spindle checkpoint mechanism to prevent homologous chromosomes from separation. The inhibition of *Chk1* expression could accelerate the occurrence of GVBD, while it had no effect on oocytes after GVBD [59]. As a downstream effector molecule in the p53 and ATM pathways induced by DNA damage in somatic cells, the presence of Chk1 indicated that the oocytes were likely to cope with the DNA damage. Thus, it was speculated that there existed DNA damage during meiotic resumption to meiosis I in oocytes, where Chk1 may be an effector molecule that the oocytes responded to the DNA damage. Similarly, when fully grown oocytes were exposed to neocarzinostatin, which can induce DNA DSBs, the mouse oocytes were arrested at MI [46]. As an effective mechanism of DNA damage response cannot be established in oocytes arrested at prophase, the subsequent two metaphase divisions may be the only possible defenses against DNA damage.

## 4. Materials and Methods

### 4.1. Reagents

All chemicals and media were obtained from Sigma-Aldrich (Shanghai, China) unless otherwise indicated.

### 4.2. Overexpression or Interference Experiments of p21 at Germinal Vesicle (GV) Stage in Bovine Oocytes

#### 4.2.1. Amplification of the Expression Vector for *p21* and Transfection of Plasmids into Hela Cells

In this experiment, the vector “Venus” with the T7 promoter and the Venus (a Yellow fluorescent variant) gene was used, which can express the Venus fluorescence protein [60,61]. The eukaryotic expression vector “*p21*-Venus” was kept in our laboratory and its fusion protein was “*p21*-Venus”. The full-length coding sequence for bovine *p21* (NM_001098958.1) was amplified from GV-stage oocytes (Figure S1C). The primer sequences were designed on the basis of Primer-BLAST (https://www.ncbi.nlm.nih.gov/tools/primer-blast/index.cgi?LINK_LOC=BlastHome); the PCR (polymerase chain reaction) data are shown in Figure S1A. The coding region sequence of *p21* was linked by restriction enzyme sites of Hind III and KpnI to the plasmid “Venus” (Figure S1B). The primer sequence synthesis and sequencing were carried out by Sangon Biotech (Shanghai, China). The sequences were blasted in NCBI.

To further test whether the recombinant plasmid *p21*-Venus was successfully constructed, HeLa cells kept in our laboratory were cultured for transfection experiments. Cells were cultured in Dulbecco’s modified Eagle’s medium (DMEM); Gibco, Shanghai, China) supplemented with 10% FBS (fetal bovine serum; HyClone, Shanghai, China) and 2 mM L-glutamine to 70% confluency in 96-well plates. Then, *p21*-Venus plasmids were transfected into HeLa cells by LipofectamineTM 2000 (Invitrogen, Beijing, China), in accordance with the manufacturer’s procedure. Simply, transfection complexes were prepared as follows: We diluted 250 ng *p21*-Venus in 25 μL Opti-MEM™ I Medium per tube and 1 μL Lipofectamine™ stem reagent in 25 μL Opti-MEM™ I Medium per tube. Then, diluted *p21*-Venus was added to diluted Lipofectamine™ stem reagent (1:1 ratio), which were incubated in parallel for 10 min at room temperature, then added into 96-well plates (100 μL/well, *p21*-Venus: 250 ng/100 μL, Lipofectamine™ stem reagent: 1 μL/100 μL). The 96-well plates were incubated at 37 °C under 5% CO_2_ for 5 h. Then, the medium was changed with fresh DMEM and cells were harvested after 24 h, in order to determine the transfection efficiency of *p21*-Venus plasmids by the green fluorescence observed under a fluorescence microscope (Leica Camera AG, Solms, Germany).

#### 4.2.2. Oocyte Collection and In Vitro Maturation (IVM)

Animal care and handling were performed according to Animal Research Committee policies of the Institute of Zoology, Chinese Academy of Sciences. Specifically, bovine ovaries were obtained from a local slaughterhouse and transported to the laboratory at 20–24 °C within 6–10 h after slaughter in a thermo-flask containing 0.9% saline solution (NaCl, 0.9% wt:vol) supplemented with penicillin and streptomycin. Follicles with a diameter ranging from 3–8 mm were aspirated using a syringe with a 12-gauge needle. The follicular fluids were pooled in a sterile centrifuge tube, and the collected cumulus–oocyte complexes (COCs) were dispersed into a 90 mm cell culture dish after three washes using COCs wash solution (TCM199 containing 10% PBS, 2.2 g/L sodium bicarbonate, 10 mmol/L HEPES; pH 7.2–7.4) with 50 μM roscovitine pre-warmed to 37 °C. Only COCs surrounded by a minimum of three cumulus cell layers and having evenly granulated cytoplasm were selected for IVM. Then, the COCs were washed 3 times in maturation medium [TCM199 containing 10% BSA, 2.5 μg/mL sodium pyruvate, 2 μmol/mL glutamine, 10 μL/mL ITS, 0.1 IU/mL hMG, 1 μg/mL E2 (Estradiol), 50 ng/mL EGF (epidermal growth factor) and 50 μg/mL uracil] without roscovitine and cultured in 100 μL of roscovitine-free maturation medium with mineral oil under 5% CO_2_ at 38.5 °C after three washes in maturation medium. The maturation medium was equilibrated in a CO_2_ incubator for 2 h at least before commencing maturation culture.

#### 4.2.3. Preparation of *p21*-Venus cRNA and *p21*-Morpholino

Venus and *p21*-Venus cRNA were transcribed in vitro from the vector (Venus) and recombinant plasmid (*p21*-Venus) using the mMESSAGE mMACHINE T7 Ultra Kit (Ambion, Inc. Life Technologies Corporation). The cRNAs (200 ng/μL) dissolved in nuclease-free H_2_O were microinjected into GV-stage oocytes to overexpress *p21*. The concentrations and optical density (OD of *p21*-Venus and Venus are shown in Table S1. The *p21*-Morpholino (5′-GTCCCTGGACAGCTCAGACATGGCA-3′) and Control Morpholino (meaningless interference segments, 5-′ACGGTACAGACTCGACAGGTCCCTG-3′) were designed and synthesized by Gene Tools LLC (Philomath, OR, USA) [62,63,64]. The control Morpholino sequence was a nonspecific and nontoxic Morpholino sequence, in contrast to the *p21*-Morpholino sequence.

#### 4.2.4. Microinjection into GV-Stage Oocytes

The COCs collected from the ovaries were cultured in a maturation medium containing 50 μM roscovitine in order to be arrested in GV-stage. After 8 h of incubation with roscovitine at 38.5 °C, the cumulus cells around oocytes were removed with 0.3% hyaluronidase. Then, denuded oocytes (Dos) at the GV-stage were microinjected. Briefly, Dos were transferred to 20 μL of operating [TCM199 containing 10% FBS, 7.5 mg/L CB (cytochalasin B)] droplets covered with mineral oil. Then, the microinjected oocytes were anchored with a fixed needle, and 1–2 μL of samples were inhaled in the injection needle to be injected into the cytoplasm using a Leica micromanipulation system (Leica Camera AG, Solms, Germany) and an Eppendorf FemtoJet microinjection system (Hamburg, Germany). The number of oocytes injected into each group was about 25–30, where the volume of samples injected into each oocyte was 10pL. After microinjection, the oocytes were washed three times with fresh maturation medium without roscovitine to completely remove the roscovitine residues. Finally, the oocytes were transferred into fresh maturation media without roscovitine under 5% CO_2_ at 38.5 °C and incubated for the next 3 h or 22 h.

To observe the location of *p21* in oocytes, the cRNAs of Venus and *p21*-Venus were microinjected into Dos at GV-stage. Following 3 h of culture, the oocytes were imaged by fluorescence microscopy (Axiocam MRm; Carl Zeiss, Shanghai, China).

To assess the efficiency of overexpression and interference of *p21* in oocytes, *p21*-Venus cRNA and *p21*-Morpholino were co-injected. Following 3 h of culture, the oocytes were imaged by fluorescence microscopy (Axiocam MRm; Carl Zeiss, Shanghai, China).

To investigate the effects of *p21* on maturation, we also used *p21*-Venus cRNA for the overexpression of p21 protein synthesis and oligonucleotide interference technique for the interference of p21 protein synthesis (the *p21* Morpholino) in oocytes. After 22 h of culture, the first polar body (PBI) excretion (PBEI) was assessed. This group of oocytes was used to study the effects of *p21* upregulation or downregulation on in vitro maturation. The oocytes were divided into five groups, as shown in Table 1.

### 4.3. Zeocin-Induced DNA Damage in Bovine Oocytes

#### 4.3.1. HeLa Cell Viability Assay

In order to delineate the suitable concentration of Zeocin (Invitrogen, Beijing, China) inducing DNA DSBs in bovine oocytes, we performed pre-experiments in HeLa cells. The cell viability calculated by the Cell Proliferation Kit I (MTT) was determined to explore the concentration range of Zeocin. Cell viability was determined by a colorimetric assay based on the ability of viable cells to metabolize 3-(4,5-dimethylthiazol-2-yl)-2,5-diphenyltetrazolium bromide. According to the specification, HeLa cells were seeded at a density of 1 × 10^4^ cells/mL into 96-well culture plates at 37 °C under 5% CO_2_. After culturing for 3 h, the cells were treated with different concentrations of Zeocin (2, 1, 0.5, 0.25, 0.125, 0.0625, 0.03125 and 0.015625 μg/μL) for 17 or 24 h. MTT (5 mg/mL) was added to each well, followed by incubation at 37 °C for 4 h. Then, the resulting formazan crystals were dissolved in DMSO. The absorbance was measured using a fluorescence microplate reader (Infinite M200, Shanghai, China) at 460 nm.

#### 4.3.2. Immunofluorescence Labeling

Zeocin is a DNA DSBs reagent. Several concentration gradients were set up to investigate the effects of Zeocin on DNA DSBs in oocytes. The bovine COCs collected were cultured in mature culture medium with 0, 10, 50, 351.68 or 1758.38 μM Zeocin (Figure S2B) for 2 h, in order to induce DNA DSBs, which were detected by immunofluorescence staining for γH2AX.

Immunofluorescence reagents were all purchased from Beyotime Biotechnology (Beyotime Biotechnology, Shanghai, China). To label the γH2AX, briefly [62,65], oocytes were fixed in 4% paraformaldehyde (PFA) at room temperature for 40 min, followed by permeabilization in 0.5% Triton X-100 for 20 min. After blocking in 1% BSA for 1 h, the oocytes were incubated with γH2AX antibody (Abcom, Shanghai, China) at 4 °C overnight, washed three times and incubated with the second antibody at room temperature for 1 h. Then, the oocytes were stained with Hoechst 33,342 (10 μg/mL) for 5 min. Finally, the oocytes were observed under a fluorescence inversion microscope (Axiocam MRm; Carl Zeiss, Shanghai, China).

#### 4.3.3. RNA Extraction and qRT-PCR

In order to explore the possible mechanisms affecting the maturation of oocytes, bovine COCs were incubated in four different concentrations of Zeocin gradients (10, 50, 351.68 or 1758.38 μM) in order to induce DNA DSBs. COCs collection referred to the above method. After 2 h of culturing in Zeocin, the oocytes were washed 3 times with mature culture media without Zeocin and cultured in mature culture medium without Zeocin for another 20 h. At 22 h, 10 immature or mature oocytes obtained after the removal of cumulus cells were used to detect the mRNA expression level of genes by qRT-PCR. The primer sequences of genes were designed on Primer-BLAST (https://www.ncbi.nlm.nih.gov/tools/primer-blast/index.cgi?LINK_LOC=BlastHome), where *β-actin* was used as a housekeeping gene (Table S2).

In order to explore the effects of cumulus cells on the response of COCs to DNA DSBs in the prophase of meiosis I, bovine COCs harvested from the ovaries were randomly divided into two groups. One group was the Dos from COCs whose cumulus cells were stripped, while the other group was COCs. Oocytes of the two groups were cultured in a mature culture medium containing 0, 10 or 50 μM Zeocin for 2 h. Similarly, 10 Dos or COCs selected randomly from the 6 groups were processed as described above in order to obtain cDNAs to detect the expression of genes (Table S2) by qRT-PCR.

A total of 10 oocytes were harvested for RNA extraction using acidic Tyrode’s solution (pH 2.5) and lysates (DTT 5 mmol/L, RNAse 20 U/mL, NP-40 1%, in Nuclease-free Water) [66]. Briefly, the selected oocytes were placed in acidic Tyrode’s solution for 20–60 s to remove the zona pellucida, then transferred to lysates (10 μL) and placed in a −80 °C freezer for at least 15 min. Next, cDNAs were obtained by SuperScript^®^ III One-Step RT–PCR System with Platinum^®^ Taq high fidelity DNA polymerase (Invitrogen, Beijing, China) to detect the expression of genes, according to the AceQ qPCR SYBR^®^ Green Master Mix (Vazyme Biotech, Nanjing, China) by qRT-PCR (95 °C 5 min; 95 °C 10 s, 60 °C 30 s, Reps 40; 95 °C 15 s, 60 °C 60 s, 95 °C 15 s).

### 4.4. IVM and Gene Expression in Oocytes Treated with ATM-Specific Inhibitor

#### 4.4.1. IVM of Oocytes with ATM-Specific Inhibitor

The collected COCs were cultured in mature culture media containing 0, 5, 8.34 or 10 μM ATM-specific inhibitor KU-55933 for 22 h. Then, the cumulus cells around oocytes were stripped by incubation in 0.3% hyaluronidase solution, and the rate of PBEI (PBEI%) was assessed.

#### 4.4.2. RNA Extraction and qRT-PCR

Subsequently, 10 mature or immature oocytes were selected randomly to carry out cDNA synthesis for gene expression detection by qRT-PCR. The mature oocytes were with PBEI, while immature oocytes were at GV-stage. RNA purification and qRT-PCR referred to the above method.

### 4.5. Statistical Analysis

Oocyte maturation experiments were repeated more than 3 times, and at least 30 oocytes were treated per experiment. The GraphPad Prism software was used to perform all statistical analyses, which was carried out by one-way analysis of variance (ANOVA), where a *p*-value less than 0.05 was considered significant.

## Figures and Tables

**Table 1:** Microinjection into germinal vesicle (GV)-stage oocytes for overexpression or interference of *p21*.

Group	Samples Microinjected
Overexpression	*p21*-Venus group: 200 ng/μL *p21*-Venus cRNA
	Venus group: 200 ng/μL Venus cRNA
Interference	*p21*-Morpholino group: 2 mmol/L *p21*-Morpholino
	Control Morpholino group: 2 mmol/L Control Morpholino
Control	nuclease-free H_2_O group: nuclease-free H_2_O

## Supplementary Materials

The following are available online at https://www.mdpi.com/1422-0067/21/23/8892/s1.
