# Supplementary material for: Coping with DNA Double-Strand Breaks via ATM Signaling Pathway in Bovine Oocytes
Source: Int J Mol Sci. 2020 Nov 24;21(23):8892. doi: 10.3390/ijms21238892 (PMC7727702; doi:10.3390/ijms21238892)
Supplement: Supplementary file 1 [file ijms-21-08892-s001.pdf]

International Journal of *Molecular Sciences*

MDPI

Article

Coping with DNA Double-Strand Breaks via ATM Signaling Pathway in Bovine Oocytes

Lili Wang [1], Xiaolei Xu [1], Mingming Teng [1], Guimin Zhao [2] and Anmin Lei [1,*]

[1] Shaanxi Stem Cell Engineering and Technology Research Center, College of Veterinary Medicine, Northwest A&F University, Yangling 712100, China; iamwanglili@nwafu.edu.cn (L.W.); xuxiaolei@nwafu.edu.cn (X.X.); tengmingming@nwafu.edu.cn (M.T.)

[2] Key Laboratory of Infection and Immunity of Shandong Province, Department of Immunology, School of Biomedical Sciences, Shandong University, Jinan 250012, China; zhaoguimin@sdu.edu.cn

* Correspondence: anminleiryan@nwsuaf.edu.cn; Tel./Fax: +86-029-87080068

Received: 24 September 2020; Accepted: 20 November 2020; Published: date

Supplementary Information

1. Identification of p21-Venus

The amplification of *p21* was carried out via PCR (Figure S1.A/C), where gel electrophoresis indicated that the coding sequence of *p21* was 500 bp (Figure S1.C) and that of the Venus vector was 6100 bp (Figure S1.B). The sequence of *p21* target fragment blasted in NCBI was correct.

A. Primer sequences and corresponding RT–PCR product size and annealing temperature [1,2].

Gene	GeneBank No.	Primer Sequence (5′–3′)	Product Length (bp)	Tm (°C)
p21	NM_001098958.1	F: ATCGAAGCTTACAGGTGCCATGTCTGAGCTGT<br>R: ATCGGGTACCGCGGGCTTCCTCCTGGAGCAGAT	500	60

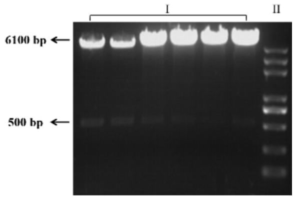

B. Identification of *p21*-Venus by restrictive enzyme digestion. I: *p21*-Venus digested by Hind III, KpnI; II: 5000 DNA marker.

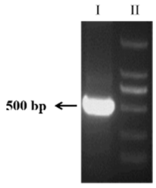

C. Product of bovine *p21* gene by RT–PCR. I: PCR product of *p21* gene; II: 2000 DNA marker.

Figure **S1**. Identification of *p21*-Venus (A/B/C).

2. Transcription In Vitro

Table S1. Concentration and OD detection of Venus and *p21*-Venus cRNA.

cRNA	Concentration (ng/μL)	A260/280	A260/230
p21-Venus cRNA	356	2.29	2.26
Venus cRNA	679	2.30	2.24

3. Effects of DNA DSBs on the Proliferation in HeLa Cells

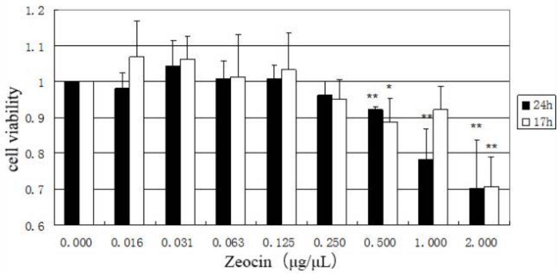

A. Relative growth rates of HeLa under Zeocin treatment. The relative values of cell activity after calculation, with significance compared to the control. Each group was were repeated 5 times.

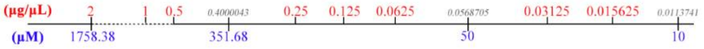

B. The conversion relationships of mass concentration (μg/μL, red) and molarity (amount of substance concentration, μM, blue) of Zeocin in the experiment. Zeocin conversion relationships (1758.38 μM ≈2 μg/μL, 351.68 μM ≈0.4000043 μg/μL, 50 μM ≈0.0568705 μg/μL and 10 μM ≈0.0113741 μg/μL).

Figure **S2**. Effects of DNA DSBs on the proliferation of HeLa cells (A/B).

4. Primer Sequences for qRT-PCR

Table S2. Primer sequences for qRT-PCR.

Gene	Primer sequence (5′–3′)
β-actin	F:TCCTCCCTGGAGAAGAGCTA<br>R:GTAGAGGTCCTTGCGGATGT
ATM	F:CTTAGGAGGAGCTTGGGCCT<br>R:CCGCTGTGTGGCAAACC
p21	F:CTAAGTGGGCAAATATGGGTCTGG<br>R:CAGGATGCTACAGGAGCTGGAAG
p53	F:AAGAAGTTGGAGCACATGACGGAG<br>R:AGAGTCGATCTCGGGGGACTCAT
RAD51	F:ATGCACCGAAGAAGGAGCTAAT<br>R:ATGCACCGAAGAAGGAGCTAAT
BRCA1	F:ACAAAGCAGCAGACACAATCTCA<br>R:TCATGGTCTCCCACACTGAAATA
Ku70	F:AATTGACTCCTTTTGACATGAGCAT<br>R:AATTGACTCCTTTTGACATGAGCAT
GDF-9	F:AGCGCCCTCACTGCTTCTATAT<br>R:TTCCTTTTAGGGTGGAGGGAA
BMP-15	F:ATCATGCCATCATCCAGAACC<br>R:TAAGGGACACAGGAAGGCTGA
FSHR	F:AATCTACCTGCTGCTCATAGCCTC<br>R:TTTGCCAGTCGATGGCATAG
H1ffo	F:CCCAAGAAGCCGAGTGAGTC<br>R:CTTGGTATCTGCTTGGCGGC
Cyclin B	F:GGAACTCACTATGCTGGACTACG

R:GCACAACAACGAGAAGGGATT

References

1. Zhao, G.-M.; Yang, W.-L.; Wu, S.-J.;Yun, Y.; Lei, A.-M. Detection for p21 Gene Expression in Bovine Oocytes Maturation and Construction of Eukaryotic Expression Vector pVenus-P21. *Acta Vet. Zootech. Inica*. **2011**, *42*, 1071–1080.
2. Zhao, G.-M. *Effect of P21 on Bovine Oocytes Meiotic Maturation*. Yangling: Northwest Agriculture and Forestry University, **2011**, 1-90.

Publisher's Note: MDPI stays neutral with regard to jurisdictional claims in published maps and institutional affiliations.

© 2020 by the authors. Submitted for possible open access publication under the terms and conditions of the Creative Commons Attribution (CC BY) license (http://creativecommons.org/licenses/by/4.0/).
